# Shoot/Root Interactions Affect Soybean Photosynthetic Traits and Yield Formation: A Case Study of Grafting With Record-Yield Cultivars

**DOI:** 10.3389/fpls.2019.00445

**Published:** 2019-04-09

**Authors:** Yanli Du, Qiang Zhao, Shengyou Li, Xingdong Yao, Futi Xie, Mingzhe Zhao

**Affiliations:** ^1^Soybean Research Institute, Shenyang Agricultural University, Shenyang, China; ^2^Institute of Crop Research, Liaoning Academy of Agricultural Sciences, Shenyang, China

**Keywords:** grafting, root physiology, grain yield, yield components, photosynthesis

## Abstract

Improvement of soybean [*Glycine max* (L.) Merr.] yield and photosynthesis physiology have been achieved over decades of cultivar breeding. Identification of the mechanisms involved in shoot-root interactions would be beneficial for the development of yield improvement breeding strategies. The objectives of this study were to investigate soybean shoot-root interactions with different-year released soybean cultivars and to evaluate their effects on grain yield and yield components. Soybean grafts used in this study were constructed with two record-yield cultivars Liaodou14 (L14) and Zhonghuang35 (Z35) and eleven cultivars released in 1966–2006 from the United States and Chinese. The grafting experiments were conducted as pot-culture experiments and repeated in 2014 and 2015. Our results showed that net photosynthesis rate (*P*_N_) was positively correlated to both root activity and root bleeding sap mass (RBSM) during the R6 reproductive stage. Moreover, different year-released soybean shoots had all exhibited capabilities of changing the root activity and architecture of L14 and Z35 rootstocks to “generation”-specific patterns during all reproductive stages. However, these influences were independent of the photosynthetic strength. Yield analysis had demonstrated that high-yielding root systems (L14 and Z35 rootstocks) could cause more than 15% of yield increase in seven out of eleven common scions in a scion-genotype-dependent manner. For Williams-descendant cultivar scions, L14 and Z35 rootstocks promoted yields mainly by increasing the seed number (SN), but those scions of Amsoy-descendent cultivars showed mainly seed weight (SW) increases when grafted onto L14 and Z35 rootstocks. On the other hand, although most tested common rootstocks did not show significant influence over the final yields in record-yield L14 and Z35 scions, they were obviously capable of shifting the formation of yield components when compared to L14 and Z35 self-grafting controls. Taken together, soybean shoots could influence the root physiology and played a crucial role in the determination of yield potentials. Synergistically with shoots, soybean roots played a more supportive role during the realization of yield potentials through root activities and by balancing the formation of yield components. These findings provided interesting insightful information for developing new breeding strategies which aim to pyramid elite physiological and yield traits by selecting specific parental combinations.

## Introduction

In eighty years between 1928 and 2008, soybean yield has been improved by 16.8 kg/ha per year for cultivars released in the maturity groups IV through V (Rogers et al., 2015). To achieve this yield improvement, the photosynthesis has been enhanced in soybean breeding due to a positive correlation between yield and photosynthetic rate (Wells et al., 1982; Boerma and Ashley, 1988; Ainsworth et al., 2012). As reported by Jin et al. (2010), the photosynthetic rates of Chinese soybean cultivars increased by 0.59% per year from 1950 to 2006. However, a recent study showed that photosynthetic capacities of more recently released cultivars have not been significantly improved compared with the older cultivars (Koester et al., 2016). Considering the fact that the photosynthetic rates of modern cultivars require higher soil nutrition conditions than older cultivars, the root systems might be potential limiting factors for the improvement of shoot photosynthetic capacities, which have rarely been considered during breeding efforts. A recent study has showed that the root functions were improved over time from 1923 to 2009 in released Chinese soybean cultivars, in which the root growth vigor correlates positively with the net photosynthetic rates (Cui et al., 2015). Recently, Li et al. (2017b) indicated that the improvement of root physiological functions was beneficial to certain shoot photosynthetic traits. Since empirical breeding techniques that have been employed traditionally mainly focused on the above-ground characteristics of cultivars, it is necessary to consider coordinating the improvements of shoot and root function toward achieving extra yield gains.

Variations of grain yield mainly can be influenced by the utilization and distribution of carbohydrates and nutrient substances during the grain filling periods. The term of a “source-sink” relationship was often used to identify and assess the limitation of grain yield either by the demand (i.e., sink strength) or by the supply (i.e., source capacity) of assimilates during the seed filling stage (Tollenaar and Aguilera, 1992). In soybean, the source capacity has been specifically defined by the production of assimilates from shoot photosynthesis during the seed filling stage (Board et al., 1995). And the sink strength has been determined by the capacity of growing seeds to absorb available assimilates. Soybean grain yield has been reported to be source-limited for older cultivars and sink-limited for newer cultivars (Liu et al., 2006). Up to date, the genetic basis underlying soybean source-sink relationships and yields including yield components still remain unknown.

In grain crops, the final yield is a complex agronomic trait and usually analyzed in terms of two primary yield components: seed weight (SW) and seed number (SN) per unit area (Board and Tan, 1995; Kahlon et al., 2011). Soybean yield can be assessed with the product of SN and SW (Roekel et al., 2015). In general, it is an efficient strategy to increase soybean seed number to gain high yield, as the number of seeds constitutes most yield variations (Slafer, 2003; Rotundo et al., 2012). The SN is continuously determined during soybean reproductive growth stages from the beginning of flowering (R2 stage) to the initiation of the seed filling stage (between stages R5 and R6) (Fehr and Caviness, 1977; Egli and Zhen-wen, 1991; Board and Tan, 1995; Jiang and Egli, 1995). Photosynthesis correlates with SN during the reproductive growth periods as the main plant “sink” capacity is composed by developing seeds. Previous studies have suggested that extending the reproductive phases, such as, prolonging the photoperiod after the pod-setting period, could increase the SN (Kantolic and Slafer, 2001, 2005, 2007; Kantolic et al., 2013). Seed weight (SW per hundred seeds) is another important soybean yield component and also an advantageous trait for the vigorous early establishment of young seedlings. SW is determined by seed size (SS) potential and the degree of seed filling. It is a trait of relatively stable heritability under fixed environmental conditions. Therefore, soybean SW is mainly influenced by environmental changes and source-sink relationships (Liu et al., 2006). An appropriate temperature as well as sufficient water and fertilization during the seed filling stage are essential to increase SW (Dornbos and Mullen, 1991; Gan et al., 2003). Moreover, soybean SW is a quantitative trait controlled by multi-genes with additive and epistatic effects (Fasoula et al., 2004; Zhang et al., 2015). Many quantitative trait loci (QTLs) have been shown to be associated with soybean SW (SoyBase, http://www.soybase.org/). Recently, Zhang et al. (2015) identified thirty-nine annotated SW candidate genes which are associated with biological processes as well as cellular and molecular functions, indicating that the determination of SW was very complex.

Although to obtain higher grain yields has always been the ultimate goal for all soybean breeders, different breeders tend to select cultivars specifically either with higher SW or more SN according to their preferences. This selective bias is often based on an acknowledged SW-SN trade-off relationship since only limited resources are available for distribution between yield-components during the seed set period (Sadras, 2007; Gambín and Borrás, 2010). To tackle the pressing challenge of meeting the needs of a growing global population (Ray et al., 2013), integration of superior SW and SN traits should provide a viable strategy for exploiting extra crop yielding potentials particularly in soybeans.

Grafting is a very useful technique that has been widely applied in plant shoot-root signaling and nutrient cross-talking researches (Matsuoka et al., 2016; Ostendorp et al., 2016). For example, grafting has been applied in regulating tree vigor, fruit sizes and quality in apple, pear and plum (Webster, 2002). Another study has also used grafting to confirm the regulatory roles of legume shoots and roots in nodule development and host/rhizobia specificity (Lohar and VandenBosch, 2005). In addition, a study in Arabidopsis using reciprocal grafting has demonstrated that the flowering can be induced by the long-distance transport of Flowering Locus T (FT) protein which moves from the leaf to the apex (Corbesier et al., 2007). Grafting has also been adopted in soybean physiological researches. Recently, Li et al. (2017a,b) reported that the photosynthesis, yield, and 100-seed mass of current-cultivar soybean scions could be improved when grafted onto rootstocks of higher root physiological activities. But, these shoot improvements were failed to be achieved with older-cultivar scions. These results suggested that the soybean root functional improvement could indirectly promote shoot photosynthesis and yield traits. Since the shoot-root interaction is fundamental to plant development and growth, a more detailed and broader understanding on soybean yield formation should be examined from shoot-root interaction perspectives. In this sense, a good understanding of the physiological effects on yielding by soybean shoot-root interactions would be a central knowledge and beneficial to soybean breeding and production.

**FIGURE 1:**
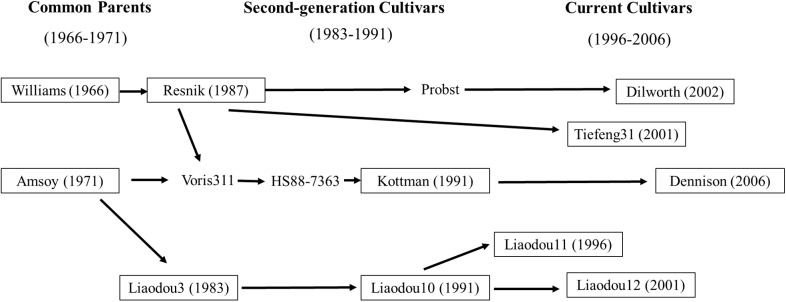
Genetic relationship of soybean cultivars in this study (boxed).

To take advantages of complicated pedigrees in the soybean germplasm, the present study collected eleven cultivars released in different decades from 1966–2006 in Liaoning, China and Ohio, United States including their common ancestors (Williams and Amsoy; Figure 1 and Supplementary Table S1). Additionally, Liaodou14 (L14) and Zhonghuang35 (Z35) were also selected as two record-yield cultivars with reported record yield of 4908 and 6089 kg/ha, respectively (Song et al., 2001; Jin and Wang, 2014). Moreover, Zhao et al. (2014) have reported that L14 and Z35 possess stronger root capacity of nutrient absorption compared with common cultivars. The objectives of this study were to graft those eleven common cultivars with L14 or Z35, respectively and: (1) to investigate the relationship between shoot photosynthesis and root physiology during the reproductive growth stages; (2) to assess the effects of shoot-root interactions on yield and yield components.

## Materials and Methods

### Plant Material and Grafting Treatment

Eleven maturity group III soybean cultivars which were bred from common ancestors Amsoy and Williams (Gizlice et al., 1994; Cui et al., 2000) and released in different decades from Liaoning Province of China (38.55°–42.32°N) and the Ohio state of the United States (38.45°–41.22°N) were used in this study (Figure 1 and Supplementary Table S1). For convenience, these cultivars were classified into three groups according to their releasing date as “common parents” (Dp, 1966–1971), “second-generation cultivars” (Ds, 1983–1991), and “current cultivars” (Dc, 1996–2006). The two high-yielding soybean cultivars Liaodou14 (Liaodou10 × Mercury) and Zhonghuang35 [(PI 486355 × Zheng 8431) × Zheng6062] were chosen because of their superior yield traits. Grafting controls for physiological traits and yield analyses were self-grafts of above cultivars. Grafts with scions of each cultivar group and L14 (or Z35) rootstocks were designated as Dp/L, Ds/L and Dc/L (or Dp/Z, Ds/Z, and Dc/Z). *Vice versa*, L/Dp, L/Ds and L/Dc (or Z/Dp, Z/s and Z/Dc) represented grafts with L14 (or Z35) scions and rootstocks of three groups of cultivars. D/L, D/Z, L/D and Z/D each represented the population average of all grafts under the same corresponding grafting model.

### Grafting and Growth

Average-sized soybean seeds were selected for each genotype and planted into soil pots (12 cm × 12 cm × 12 cm) on both the seventh of May, 2014 and the fourth of May, 2015. Grafting experiments were performed at 10 days after seed plantation with young soybean seedlings as described by Li et al. (2017a,b). Grafted seedlings were maintained under greenhouse temperature (23–28°C), low light intensity (approx. 360 μmol [photon] m^-2^s^-1^), and approx. 90% of relative humidity for a recovery period of 2 weeks.

Greenhouse-recovered soybean grafts were transplanted into pots (25 × 30 × 25 cm) with 12.5 kg soil per pot and grown under open-field conditions. The soil nutritional characteristics were: 16.87 g/kg soil organic matter, 0.79 g/kg total nitrogen, 0.07 g/kg available nitrogen, 0.02 g/kg available phosphorus, 0.14 g/kg available potassium, and pH 7.33. Dripping irrigation was adopted to keep the soil moist at ∼70% of the field water-holding capacity.

### Pot-Culture Experiment Design

All pot-culture experiments were performed at two experimental sites at Shenyang Agricultural University (41°82′N, 123°57′E) in Liaoning Province of China during each year of 2014 and 2015. Two grafted plants of the same grafting were grown in a single pot and considered as one experimental unit. All experiments adopted a randomized complete block design for each experimental site with three-unit replicates per grafting.

### Agronomic Trait Measurements

All measurements of soybean physiological indexes were conducted during the flowering (R2), grain-filling (R5), and the late grain-filling (R6) stages with three biological replicates.

For the measurement of photosynthetic parameters, the upper third (from the top of the main stem) fully unfolded trifoliolate leaf on each plant was selected and measured by a *LI*-*6400* portable photosynthesis system (*Li*-*Cor* *Inc*., Lincoln, NE, United States). The light-saturation point was set to 1,200 μmol (photon) m^-2^ s^-1^ to measure net photosynthetic rate. The ambient temperature of the soybean leaf was kept between 25–30°C. CO_2_ concentration was 380 μmol (CO_2_) mol^-1^, relative humidity was 60–65%, and air flow was 500 μmol s^-1^. The leaf greenness was measured with a *SPAD*-*502* leaf Chl meter (*Minolta* *Camera* *Co*., Osaka, Japan). Other photosynthetic parameters (*g*_s_, *E*) were measured with methods described by Li et al. (2017a,b). All photosynthetic measurements were conducted between 9:00 and 11:00 AM.

For the measurement of soybean root activity (RA) indexes, root bleeding sap (RBSM) samples were collected according to the method described by Peoples (1989). Briefly, the shoot was cut off at the cotyledon node, followed by the collection of bleeding sap using a centrifuge tube and absorbent cotton. The triphenyl tetrazolium chloride (TTC) method (Wang et al., 2009) with root tip tissues was used to measure RA.

For the measurement of root architecture indexes, sampled roots were cleaned with water and scanned for root length measurements on the root scanning image analysis system WinRHIZO Pro 2012b (*Regent Instruments*, *Inc*., Québec, QC, Canada). To measure root biomass per plant, root samples were oven-dried at 105°C for 30 min and kept at 85°C until the root weight remained unchanged.

For measurements of yield and yield components, plants were harvested at maturity. SN and SW per plant were measured for three biological replicates.

### Data Processing and Statistical Analysis

Data were analyzed by SPSS-17.0 (SPSS Inc., Chicago, IL, United States). The data for photosynthetic parameters and root parameters were subjected to an analysis of variance (*ANOVA*) in a general linear model (GLM) with grafting treatment as fixed effect and year as a random factor (Table 2). The data for yield and yield component when record-yield cultivars as rootstocks were subjected to an *ANOVA* in a GLM with grafting treatment and genotype as fixed effects, year as a random factor (Table 3). The data for yield and yield component when record-yield cultivars as scions were subjected to an *ANOVA* in a GLM with genotypes as fixed effects and year as a random factor (Table 4). Means were subjected to the least significant difference (*LSD*) test at the *P* < 0.05 level. The correlation between photosynthetic parameters and root parameters was assessed via *Pearson’s* product-moment correlation.

## Results

### Positive Correlation Between P_N_ and RA

To define the interactions between soybean shoot and root, correlative relationships between shoot and root physiological indexes were first examined in the grafts L14 and Z35 (Table 1 and Supplementary Table S2). Eight indexes including four photosynthesis, two root activity, and two root architecture indexes were examined for each self-graft and graftings with L14/Z35. Then, *Pearson’s* correlation analysis was conducted between each pair of photosynthetic and root indexes using population averages. The *P*_N_ exhibited a significant positive correlation with both RA and RBSM only during the late seed filling R6 stage (Table 1), regardless of differences in grafting models and experimental years. However, no robust correlation could be identified between photosynthetic and root architectural indexes (Supplementary Table S2). This positive *P*_N_ and RA correlation indicated that robust interactions on the physiology level existed between the soybean shoot and root.

**Table 1:** *Pearson’s* correlation coefficients between photosynthetic and root activity indexes during the reproductive stages of 2014 and 2015.

		Year of 2014						Year of 2015					
		R2		R5		R6		R2		R5		R6	
		RA	RBSM	RA	RBSM	RA	RBSM	RA	RBSM	RA	RBSM	RA	RBSM
D/D	*P*_N_	0.33 ^NS^	0.34^NS^	0.09^NS^	0.08^NS^	0.54 **	0.51 **	0.02^NS^	–0.05^NS^	–0.02^NS^	–0.03^NS^	0.41 *	0.44 *
	*g*_s_	–0.13^NS^	–0.29^NS^	–0.01^NS^	–0.17^NS^	0.60 **	0.64 **	0.31^NS^	–0.19^NS^	–0.09^NS^	–0.20^NS^	0.26^NS^	0.28^NS^
	*E*	0.02^NS^	–0.08^NS^	0.29^NS^	0.16^NS^	0.17^NS^	0.19^NS^	0.11^NS^	–0.09^NS^	0.31^NS^	0.18^NS^	0.04^NS^	0.02^NS^
	Leaf greenness	0.30^NS^	0.35 *	–0.10^NS^	–0.12^NS^	–0.06^NS^	–0.10^NS^	0.24^NS^	–0.15^NS^	–0.22^NS^	–0.16^NS^	0.09^NS^	0.07^NS^
L/D	*P*_N_	0.18^NS^	–0.19^NS^	0.09^NS^	0.16^NS^	0.75 **	0.70 **	0.35 *	0.05^NS^	0.06^NS^	–0.08^NS^	0.66 **	0.58 **
	*g*_s_	–0.09^NS^	0.19^NS^	–0.22^NS^	–0.29^NS^	0.73 **	0.74 **	0.13^NS^	0.26^NS^	–0.25^NS^	–0.32^NS^	0.57 **	0.60 **
	*E*	–0.11^NS^	0.14^NS^	0.184^NS^	–0.01^NS^	0.53 **	0.56 **	0.15^NS^	0.43 *	0.15^NS^	0.02^NS^	0.59 **	0.60 **
	Leaf greenness	0.21^NS^	–0.29^NS^	0.15^NS^	0.22^NS^	0.29^NS^	0.16^NS^	0.29^NS^	0.08^NS^	0.28^NS^	0.16^NS^	0.02^NS^	–0.11^NS^
Z/D	*P*_N_	–0.00^NS^	0.07^NS^	–0.30^NS^	–0.44 *	0.88 **	0.83 **	0.74 **	0.53 **	0.53 **	0.61 **	0.69 **	0.58 **
	*g*_s_	–0.17^NS^	–0.57 **	–0.17^NS^	–0.14^NS^	0.37 *	0.49 **	0.44 *	0.09^NS^	0.02^NS^	0.00^NS^	0.43 *	0.39 *
	*E*	–0.35 *	–0.39 *	0.03 ^NS^	0.06^NS^	0.57 **	0.56 **	0.40 *	–0.01^NS^	0.49 **	0.47 **	0.56 **	0.42 *
	Leaf greenness	–0.19^NS^	–0.03^NS^	–0.11^NS^	–0.10^NS^	0.13^NS^	0.09^NS^	0.53 **	0.60 **	0.41 *	0.60 **	0.07^NS^	0.03^NS^
D/L	*P*_N_	–0.09^NS^	–0.16^NS^	0.06^NS^	0.00^NS^	0.81 **	0.83 **	0.26^NS^	0.23^NS^	–0.13^NS^	–0.12^NS^	0.74 **	0.71**
	*g*_s_	–0.08^NS^	–0.29 ^NS^	–0.16^NS^	–0.17^NS^	0.63 **	0.71**	–0.00 ^NS^	–0.16^NS^	0.07^NS^	0.00^NS^	0.63 **	0.63 **
	*E*	–0.35 *	–0.42 *	–0.03^NS^	–0.08^NS^	0.43 *	0.46 **	–0.21^NS^	–0.24^NS^	0.03^NS^	0.12^NS^	0.71 **	0.74 **
	Leaf greenness	0.09^NS^	–0.02^NS^	–0.079^NS^	–0.19^NS^	0.23 ^NS^	0.27^NS^	0.16^NS^	0.07^NS^	0.03^NS^	0.12^NS^	0.04^NS^	–0.02^NS^
D/Z	*P*_N_	0.17^NS^	0.35 *	–0.18^NS^	–0.18^NS^	0.86 **	0.89 **	0.23^NS^	0.46 **	–0.22^NS^	–0.14^NS^	0.78 **	0.75 **
	*g*_s_	–0.10 ^NS^	0.19^NS^	–0.22^NS^	–0.29^NS^	0.17^NS^	0.33^NS^	–0.00^NS^	0.30^NS^	–0.28^NS^	–0.25^NS^	0.37 *	0.38 *
	*E*	–0.15^NS^	–0.02^NS^	0.22^NS^	0.10^NS^	0.63 **	0.63 **	–0.04^NS^	0.15 ^NS^	0.02^NS^	–0.00^NS^	0.62 **	0.56 **
	Leaf greenness	0.12^NS^	0.36 *	0.09^NS^	0.13^NS^	0.12^NS^	0.13^NS^	0.01^NS^	0.26^NS^	0.02^NS^	0.07^NS^	0.15^NS^	0.14^NS^


R2, the flowering stage; R5, early grain-filing stage; R6, late grain-filling stage. D, mean of eleven common cultivars; D/D, self-grafts; L/D or Z/D, grafts with L14/Z35 scions; D/L or D/Z, grafts with L14/Z35 rootstocks. *P*_*N*_, net photosynthetic rate; *g*_*s*_, stomatal conductance; *E*, transpiration rate. RA, root activity; RBSM, root bleeding sap mass. The bolded values highlighted the significant correlations of PN with RA and RBSM, respectively under different grafting combinations at R6 stage. *P < 0.05, **P < 0.01, NS, not significant.

### RA Positive Influences on P_N_

Table 2 summarizes the mean square of each factor (2 years and three genotypes including two parent cultivars, four second-generation cultivars and five current cultivars) and their interactions for physiological parameters of shoot and root among different grafting combinations. There was a significant effect of grafting combinations on *P*_N_, RA, and RBSM. However, no significant effect of year on these physiological parameters of shoot and root. Therefore, we used a two-year average of these parameters in this study.

**Table 2:** Analysis of variance in physiological parameters involved in shoot and root under different grafting combinations.

	Source of variations	R2	R5	R6	R2	R5	R6	R2	R5	R6
				
		D/D			L/D			Z/D		
*P*_N_	Genotypes (G) (df = 2)	0.24^NS^	5.42^NS^	33.32*	1576.02**	1830.10**	24.98*	5320.18**	1686.69**	65.20*
	Year (Y) (df = 1)	2.36^NS^	0.95^NS^	1.89^NS^	1.00^NS^	0.08^NS^	0.39^NS^	0.70^NS^	0.02^NS^	0.08^NS^
	G × Y (df = 2)	4.85*	0.88^NS^	0.41^NS^	0.00^NS^	0.00^NS^	2.41^NS^	0.00^NS^	0.00^NS^	2.28^NS^
			D/D			D/L			D/Z	
RA	Genotypes (G) (df = 2)	5.64^NS^	77.52*	65.09*	831.67**	172.43**	68.89*	852.73**	63.47*	547.60**
	Year (Y) (df = 1)	0.00^NS^	3.00^NS^	9.87^NS^	1.71^NS^	3.44^NS^	0.00^NS^	1.68^NS^	0.24^NS^	2.69^NS^
	G × Y (df = 2)	44.31**	3.11^NS^	1.68^NS^	0.12^NS^	0.88^NS^	1.74^NS^	0.13^NS^	2.10^NS^	0.22^NS^
RBSM	Genotypes (G) (df = 2)	2.50^NS^	209.90**	160.76**	36.20*	48.26*	41.25*	83.00*	50.10*	1357.36**
	Year (Y) (df = 1)	0.63^NS^	0.32^NS^	4.25^NS^	0.07^NS^	8.15^NS^	2.01^NS^	0.21^NS^	7.47^NS^	0.71^NS^
	G × Y (df = 2)	20.26**	0.75^NS^	0.43^NS^	0.71^NS^	3.46*	2.30^NS^	0.30^NS^	3.52*	0.06^NS^
Root length	Genotypes (G) (df = 2)	10.02^NS^	78.30*	8.45^NS^	5.71^NS^	25.92*	0.41^NS^	17.60^NS^	9.69^NS^	7.38^NS^
	Year (Y) (df = 1)	0.10^NS^	0.06^NS^	0.08^NS^	1.35^NS^	0.00^NS^	0.01^NS^	3.85^NS^	4.19^NS^	1.68^NS^
	G × Y (df = 2)	2.49^NS^	0.32^NS^	2.12^NS^	2.31^NS^	0.44^NS^	11.39**	1.26^NS^	1.24^NS^	0.94^NS^
Root dry mass	Genotypes (G) (df = 2)	4.48^NS^	6.06^NS^	14.63^NS^	0.92^NS^	1.05^NS^	0.40^NS^	5.86^NS^	0.69^NS^	0.69^NS^
	Year (Y) (df = 1)	1.37^NS^	4.73^NS^	3.80^NS^	1.32^NS^	4.31^NS^	2.01^NS^	5.16^NS^	1.40^NS^	7.20^NS^
	G × Y (df = 2)	4.11*	5.50**	2.97^NS^	3.80*	4.58*	6.40**	1.26^NS^	8.80**	4.80*


D/D, self-grafts (including Dp/Dp, self-grafts of parental cultivars; Ds/Ds, self-grafts of second-generation cultivars; Dc/Dc, self-grafts of current cultivars); L/D, grafts with L14 scion (including L/Dp, parental cultivars grafted onto L14 scion; L/Ds, second-generation cultivars grafted onto L14 scion; L/Dc, current cultivars grafted onto L14 scion) (as in Z/D); D/L, grafts with L14 rootstock (including Dp/L, parental cultivars grafted onto L14 rootstock; Ds/L, second-generation cultivars grafted onto L14 rootstock; Dc/L, current cultivars grafted onto L14 rootstock) (as in D/Z). *P*_*N*_, net photosynthesis rate, RA, root activity, RBSM, root bleeding sap mass, *P < 0.05, **P < 0.01, NS, not significant.

**FIGURE 2:**
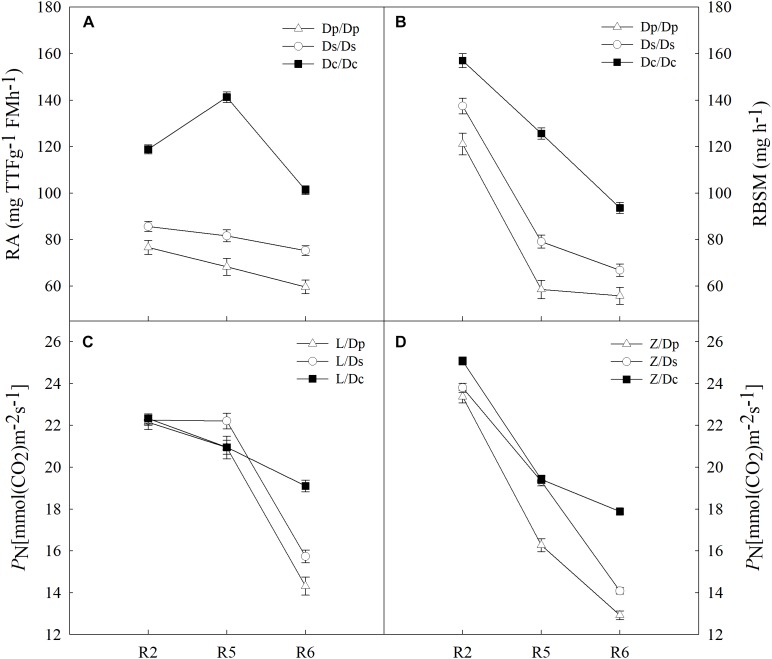
**(A)** The root activity (RA) of different self-grafts (Dp/Dp, Ds/Ds and Dc/Dc) during R2-R6 stages; **(B)** the RBSM of different self-grafts (Dp/Dp, Ds/Ds and Dc/Dc) during R2-R6 stages; **(C)** the PN of different grafts with L14 scions (L/Dp, L/Ds and L/Dc) during R2-R6 stages; **(D)** the PN of different grafts with Z35 scions (Z/Dp, Z/Ds and Z/Dc) during R2-R6 stages.

To further investigate the impacts on shoot by different root systems, L14 (L) and Z35 (Z) scions were grafted onto the parental (Dp), the second-generation (Ds) and the current (Dc) rootstocks (Figure 1 and Supplementary Table S1). Then photosynthetic properties were studied for each grafting model. With the progressing of reproductive stages, the strength of RA for all three models of grafts showed a slightly decreasing tendency with the highest value during the R5 stage (Figure 2). In addition, the average RA and RBSM of the Dc rootstocks were distinctively higher than those of Dp and Ds rootstocks for all reproductive stages (Figure 1). However, regarding to the shoot *P*_N_, no obvious difference among L/Dc, L/Dp, and L/Ds grafting models was observed during R2 and R5 stages. In addition, similar observations on *P*_N_ were also made for Z35 scions (Figure 2). These results indicated that RA exerted little impacts on shoot photosynthesis during the early reproductive stages. However, the average *P*_N_ of L/Dc (Z/Dc) was much higher than those of L/Dp (Z/Dp) and L/Ds (Z/Ds), respectively, during the R6 stage. Considering the fact that higher strength of RA and RBSM in Dc roots than in Dp and Ds roots, it was reasonable to postulate that stronger root activities could support higher photosynthetic capacity during late reproductive stages of soybean. Furthermore, when other photosynthetic indexes were subjected to the same examination, no significant association with RA strength was identified (data not shown).

### Shoot Influences on RA and Root Architecture

**FIGURE 3:**
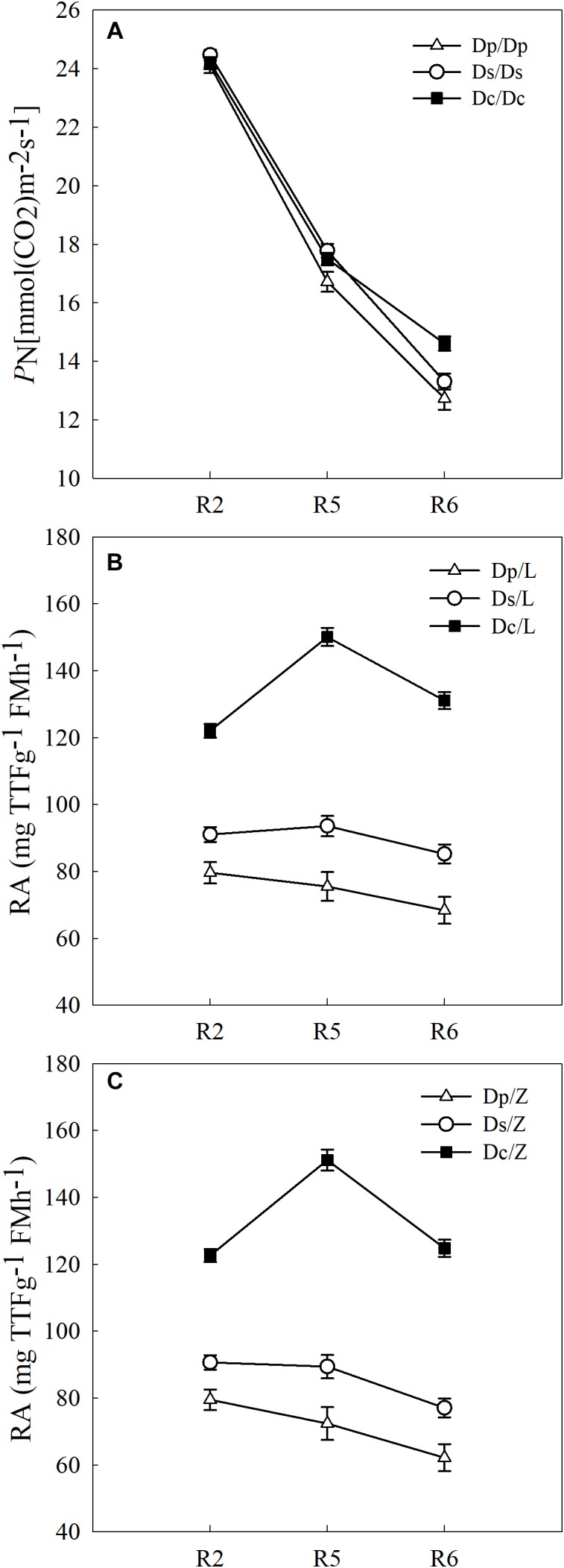
**(A)** The PN of different self-grafts (Dp/Dp, Ds/Ds and Dc/Dc) during R2-R6 stages; **(B)** the RA of different grafts with L14 rootstocks (Dp/L, Ds/L and Dc/L) during R2-R6 stages; **(C)** the RA of different grafts with Z35 rootstocks (Dp/Z, Ds/Z and Dc/Z) during R2-R6 stages.

**FIGURE 4:**
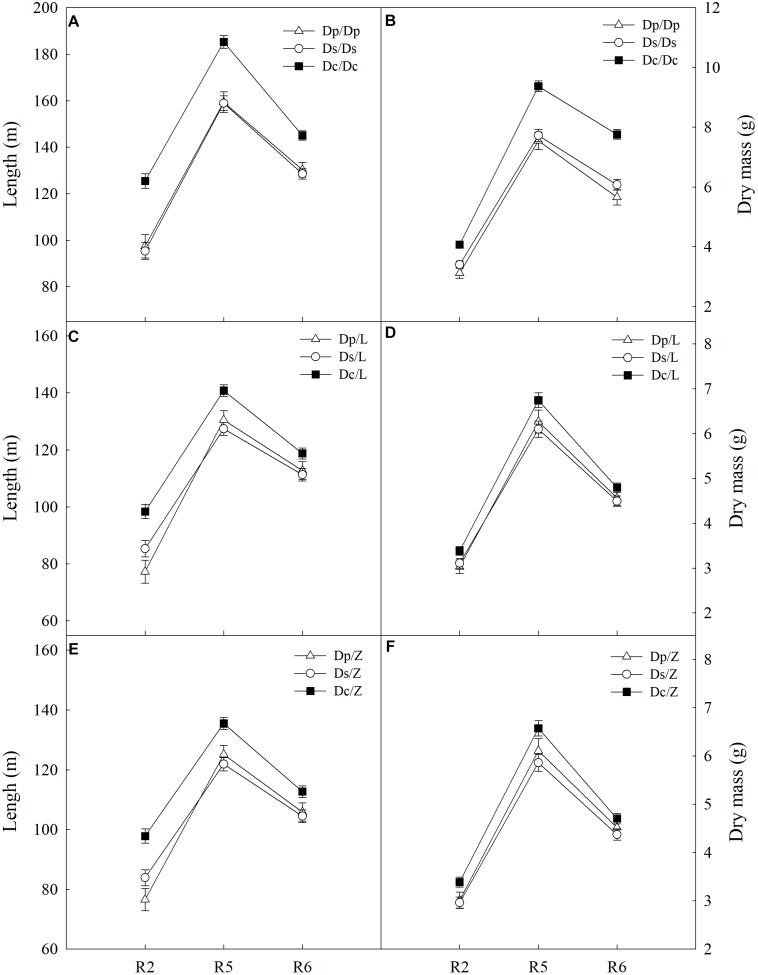
**(A)** The root length of different self-grafts (Dp/Dp, Ds/Ds and Dc/Dc) during R2-R6 stages; **(B)** the root dry mass of different self-grafts (Dp/Dp, Ds/Ds and Dc/Dc) during R2-R6 stages; **(C)** the root length of different grafts with L14 rootstocks (Dp/L, Ds/L and Dc/L) during R2-R6 stages; **(D)** the root dry mass of different grafts with L14 rootstocks (Dp/L, Ds/L and Dc/L) during R2-R6 stages; **(E)** the root length of different grafts with Z35 rootstocks (Dp/Z, Ds/Z and Dc/Z) during R2-R6 stages; **(F)** the root dry mass of different grafts with Z35 rootstocks (Dp/Z, Ds/Z and Dc/Z) during R2-R6 stages.

To answer how soybean shoot influences the root properties, grafts with Dp, Ds, and Dc scions and L14 (Z35) rootstocks were examined for RA and morphological changes. First, Dp, Ds, and Dc self-grafts showed almost indistinguishable photosynthetic characteristics in *P*_N_ (Figure 3) and other indexes (*g*_s_, *E*, and leaf greenness) (data not shown) during all tested reproductive stages, which indicated that the photosynthetic capacities of current cultivars were not significantly improved over those of parental and second-generation cultivars. Secondly, a sharp decreasing trend in photosynthetic capacities was found when soybean plants entered more mature reproductive stages, reasonable if considering the leaf senescenceing process (Figure 3). Interestingly, the L14 (or Z35) rootstocks in Dp/-, Ds/-, and Dc/- grafts showed very different levels of RA (Figure 3) and architectural characteristics (Figure 4), similar to the pattern as Dp, Ds, and Dc roots were compared (Figure 2, 4). For example, during the R5 stage, the average RA of the Dc/L graft was approximately 98% higher than Dp/L and 60% than Ds/L, respectively, while 108 and 70% when L14 rootstocks were replaced by Z35 rootstocks. In addition, the average root length of Dc/L roots was 7.8% longer than Dp/L and 10.5% longer than those of Ds/L. Moreover, the average dry mass of Dc/L roots was 7.6 and 10.6% heavier than those of Dp/L and Ds/L roots, respectively. These data suggested that the soybean shoot could notably influence both RA and architecture seemingly independent of photosynthetic strength.

### The Yield Formation of Common Shoots Influenced by High-Yield Rootstocks

*ANOVA* analysis with grafts in this study showed significant variations (*P* < 0.05) in SN and SW (seed mass per hundred seeds) among genotypes under different grafting combinations (Tables 3, 4). There was no significant effect of year on SN, however, a significant effect on seed weight was observed (Tables 3, 4). In an overview examination of all grafts for yearly relative changes in yield components in 2014 and 2015, L14 exhibited quite stable performances both as scions or rootstocks. However, grafts with Z35 scions showed notable variations between 2014 and 2015 (Tables 4, 6). Since precipitation during the soybean grain-filling period of 2014 was less compared to 2015, this unstable yearly performance of Z35 grafts could be explained by its relatively weaker environmental adaptability compared to L14, noted that Z35 was bred in Central China while L14 was bred in Northeastern China, the same region where this study was performed. This might explain the different effect of year on yield in Z35 grafting combinations (Table 4).

**Table 3:** Analysis of variance in yield, seed number and seed weight when L14 and Z35 as rootstock in 2014 and 2015.

Source of variations	L14 as rootstocks			Z35 as rootstocks		
	Yield (g)	seed number	seed weight (g)	Yield (g)	seed number	seed weight (g)
Treatment(T) (df = 1)	12723.84**	2699.05*	3283.91*	8.90^NS^	0.10^NS^	677.04*
Genotype(G) (df = 10)	13.06**	6.05**	10.24**	21.23**	5.80**	10.22**
Year(Y) (df = 1)	1.17^NS^	0.33^NS^	11.50*	7.48^NS^	0.23^NS^	11.54*
T × G (df = 10)	3.42*	2.84^NS^	3.26*	3.20*	0.78^NS^	2.2^NS^
T × Y (df = 1)	0.01^NS^	0.01^NS^	0.01^NS^	7.01*	8.56*	0.04^NS^
G × Y (df = 10)	3.25*	6.33**	3.47*	2.70^NS^	3.84*	3.06*
T × G × Y (df = 10)	2.83**	1.36^NS^	3.25**	2.16*	2.20*	4.53**


*P < 0.05, **P < 0.01, NS, not significant.

**Table 4:** Analysis of variance in yield, seed number and seed weight when L14 and Z35 as scion in 2014 and 2015.

Source of variations	L14 as scion			Z35 as scion		
	Yield (g)	Seed number	Seed weight (g)	Yield (g)	Seed number	Seed weight (g)
Genotype (G) (df = 11)	1.04^NS^	3.63*	6.86**	2.39^NS^	4.60**	6.14**
Year (Y) (df = 1)	4.07^NS^	0.03^NS^	7.67*	15.61**	1.48^NS^	5.88*
G × Y (df = 11)	7.37**	10.34**	7.81**	5.26**	7.71**	8.00**


*P < 0.05. **P < 0.01, NS, not significant.

A close examination of yield changes after grafting had shown that L14 rootstocks could increase yield by more than 15% when grafted with seven out of 11 different common scions compared to self-grafting controls. Z35 rootstocks also exhibited similar yield improving capabilities as L14 but to a slightly lesser extent (Table 5). Among the seven most yield-improved grafts, four common scions showed significant SN increases and five scions showed SW per hundred increases on L14 (comparable to Z35) rootstocks. To our surprises, the genotypes with increased SN were Kottman, Dilworth, Dennison, and Tiefeng31, which all contained bloodline from the parental cultivar of Williams (Table 5, Figure 1, and Supplementary Table S1). On the other hand, the five genotypes with increased SW were descendants of Amsoy including Dennison, Tiefeng31, Liaodou11, and Liaodou12 (Figure 1 and Supplementary Table S1) and the parental line Amsoy itself (Table 5). These results not only showed that high-yielding root systems could contribute to improving the final yield significantly but also proved that changes in yield components happened in a shoot-genotype dependent manner. Finally, since Dennison was the only current cultivar that contained bloodlines from both Williams and Amsoy, it was not surprising that it achieved increases in both SN and SW and consequently realized the highest yield gain in the Dennison/L14 graft by 32% (Table 5).

**Table 5:** Relative changes in yield formation by grafting with record-yield rootstocks in 2014 and 2015.

Traits (/Plant)	Treatment	Cultivars										
		Williams	Amsoy	Liaodou10	Liaodou3	Resnik	Kottman	Dilworth	Dennison	Tiefeng31	Liaodou11	Liaodou12
**2014**												
Yield (g)	D/L	7.42^NS^	18.56**	17.7**	8.27*	4.52*	19.86**	15.53**	29.38**	38.76**	23.26**	14.35**
	D/Z	0.94^NS^	4.98^NS^	3.73^NS^	8.42**	4.96*	9.23*	10.33*	17.35*	10.41*	15.3*	16.94*
Seed number	D/L	20.87^NS^	22.16**	5.47^NS^	5.1^NS^	–3.65^NS^	23.18*	13.04*	20.22**	13.52**	–0.43^NS^	4.91^NS^
	D/Z	3.21^NS^	7.73^NS^	–11.85^NS^	–0.44^NS^	–13.24**	7.58*	14.03**	16.07**	9.57*	–3.23^NS^	8.27^NS^
Seed weight (g)	D/L	–12.8^NS^	7.99**	1.32^NS^	3.26^NS^	6.36^NS^	7.22^NS^	5.28*	14.32**	29.94**	27.41**	16.73**
	D/Z	–1.12^NS^	14.18**	6.65*	5.47**	3.72^NS^	11.22^NS^	0.67^NS^	17.45*	27.49**	15.52**	9.65**
**2015**												
Yield (g)	D/L	1.49^NS^	18.22*	8.55^NS^	8.87*	4.62*	26.34**	23.87**	32.46**	17.01**	15.42**	15.98**
	D/Z	–1.94^NS^	17.7**	3.39^NS^	8.42*	1.68^NS^	22.88*	14.69**	22.03**	25.84*	15.85*	6.86*
Seed number	D/L	2.98^NS^	0.80^NS^	5.04^NS^	5.10^NS^	–3.65^NS^	48.14**	31.64**	20.35*	23.61*	9.05^NS^	6.37^NS^
	D/Z	11.92*	5.07^NS^	4.32^NS^	–0.44^NS^	–1.19^NS^	26.24**	23.73**	18.05*	14.7**	–3.42^NS^	–9.45^NS^
Seed weight (g)	D/L	–6.37^NS^	16.89*	5.52^NS^	3.26^NS^	12.36*	–11.19^NS^	6.62^NS^	28.82*	12.52*	10.95**	23.2**
	D/Z	–8.61**	12.58*	1.63^NS^	5.47*	2.88^NS^	0.95^NS^	3.51^NS^	20.63**	14.88**	18.67**	24.29**


Data was calculated by: (graft–self-graft) / self-graft × 100%. D/L or D/Z - the cultivars released in different decades grafted onto L14 or Z35 rootstocks, respectively. *P < 0.05, **P < 0.01, NS, not significant.

### The Yield Formation of High-Yielding Shoots Influenced by Common Rootstocks

Next, the yield formation of grafts with common rootstocks was studied to identify particularly negative impacts on high-yielding L14 (Z35) scions by different year- released common rootstocks. Contrary to our prediction, significant yield decreases were found only when high-yielding L14 and Z35 scions were grafted onto Williams and Amsoy (two parental cultivars), and the Liaodou10 rootstocks. While, the rest grafting-combinations using second-generation and current rootstocks produced only minor yield changes (Table 6). These results indicated that the record-yield L14 and Z35 shoots should possess stable yield potentials and probably also highly plastic in the realization of yield potentials by exploiting the root support to the maximum extent.

**Table 6:** Relative changes in yield formation by grafting with record-yield scions in 2014 and 2015.

Traits (/Plant)	Treatment	Cultivars										
		Williams	Amsoy	Liaodou10	Liaodou3	Resnik	Kottman	Dilworth	Dennison	Tiefeng31	Liaodou11	Liaodou12
**2014**												
Yield (g)	L/D	–12.19*	–11.65*	–6.17*	–3.38^NS^	–5.51^NS^	–5.02^NS^	–4.89^NS^	3.22^NS^	–5.12^NS^	8.26^NS^	–4.42^NS^
	Z/D	–21.9**	–28.01**	–24.46**	–13.23**	–21.55**	8.33*	–1.03^NS^	1.66^NS^	–18.44**	–7.48*	–10.47*
												
Seed number	L/D	–0.76^NS^	–6.37^NS^	–12.44*	–8.95*	–1.06^NS^	10.34^NS^	19.27**	20.48**	–5.92^NS^	–16.54**	–28.68**
	Z/D	–23.81**	–24.21**	–35.58**	–23.02**	–21.43**	19.71**	7.44^NS^	18.65**	–24.34**	–24.21**	–31.61**
Seed weight (g)	L/D	9.92*	9.33**	6.75**	8.86**	–1.37^NS^	–3.99^NS^	–13.74**	–14.59**	–10.47**	23.02**	20.55**
	Z/D	6.11^NS^	1.10^NS^	17.24**	15.44**	1.91^NS^	4.20^NS^	–7.13^NS^	–8.86^NS^	–14.29**	13.49**	12.85**
**2015**												
Yield (g)	L/D	–12.61*	–16.43**	–7.74**	–0.38^NS^	–4.38^NS^	12.25*	7.61*	4.50^NS^	–9.90*	–0.04^NS^	–1.64^NS^
	Z/D	–12.95**	–14.16**	–9.33*	3.29^NS^	–9.80**	12.69^NS^	2.90^NS^	–0.78^NS^	0.46^NS^	3.60^NS^	–6.25^NS^
Seed number	L/D	–25.18**	–29.56**	–21.78**	–13.58**	–14.43*	26.45^NS^	14.57**	10.18**	–19.10**	–26.45**	–22.77**
	Z/D	–24.75**	–25.89**	–21.62**	–7.4**	–8.96*	9.82^NS^	11.1**	8.68^NS^	–13.37*	–30.01**	–28.31**
Seed weight (g)	L/D	20.85**	23.74**	20.54**	18.49**	18.51**	–10.35^NS^	–10.02**	–10.02**	–5.38^NS^	29.14**	14.47**
	Z/D	22.24*	23.49*	18.13*	17.03*	3.07^NS^	5.61^NS^	–9.45^NS^	–11.88^NS^	–5.87^NS^	40.3**	14.91^NS^


Data was calculated by: (graft–self-graft) / self-graft × 100%. L/D or Z/D - the cultivars released in different decades grafted onto L14 or Z35 scions, respectively. *P < 0.05, **P < 0.01, NS, not significant.

Moreover, either L14 or Z35 scions on eight of eleven rootstocks displayed a clear decreasing tendency (*P* < 0.05) in SN but accompanied by enhancements in SW. Among these grafts, the Z35/Liaodou11 graft produced the highest SN reduction of 30% and SW increase of 40.3% (Table 6). These data proved that rootstocks of different year-released cultivars were capable of dramatically shifting the balance between yield components of L14 and Z35 shoots, although with relatively much smaller impact on the final yield.

L14 and Z35 were record-yield cultivars, both producing the highest SN and the lowest SW among all studied cultivars (Supplementary Figure S1). Very interestingly, Kottman, Dilworth, and Dennison were the only three genotypes which seemed to be able to maintain or even slightly improve SN in L14 and Z35 scions but caused mild SW decreases (Table 6). For instance, Dilworth rootstocks achieved ∼15 and ∼11% SN increases in L14 and Z35 shoots, respectively, in 2015. Dilworth self-grafts produced an average SN of 170 per plant, which was about 30% lower than the average SN of L14 and Z35 (Supplementary Figure S1). Therefore, this significant improvement of the already very strong SN trait in L14 and Z35 by the “weaker” Dilworth rootstock had provided a live case, from which it could be learned that the synergistic effect of the soybean shoot-root interaction might be the key to provide a SN-increase strategy and consequently to produce extra yield gain in current high-yielding soybean cultivars.

## Discussion

Crop yield is a complex agronomic trait which is influenced by multiple factors such as the canopy interception of solar radiation and the conversion efficiency of this intercepted radiation into biomass and ultimately into harvestable products (i.e., plant harvest index, HI) (Monteith and Moss, 1977; Tomeo and Rosenthal, 2017). Common strategies for the improvement of crop yield potentials typically involve two aspects: accelerating the yield potential or diminishing the yield loss (von Caemmerer and Evans, 2010; Zhu et al., 2010; Ort et al., 2015). Manipulating single targets is generally not promising to achieve dramatic yield gains; therefore, a systematic understanding of relationships among yield controlling components is necessary for the design of mechanistic strategies to achieve a notable leap in yield improvement. However, since roots are covered by soil, the contribution of root function to yield potentials still remains understudied (Cui et al., 2015; Li et al., 2017a,b). This study used the grafting to evaluate soybean shoot-root interactions and their influences on yield.

### Soybean Shoot-Root Interaction

At the whole plant level, both communication and coordination between the shoot and root are essential for plants to optimize growth and to efficiently respond to environmental fluctuations. In fact, the formation of agricultural traits (especially yield) should be viewed as comprehensive outcomes of dynamic systems and should thus be investigated from a systematic perspective. In this sense, grafting offers an excellent opportunity to dissect the shoot-root interaction by manipulating combinations of genotypes or treatments, in addition to its successful adoption in the asexual plant propagation and agricultural trait improvement. In the present study, the shoot-root interaction on the physiological level was first examined in grafting populations using commonly practiced indexes, and robust positive correlations between net photosynthetic rate and RA and RBSM was identified for the late grain filling R6 stage (Table 1).

In crops, photosynthetic capacities and the root activity generally decrease with the progressing of maturity (Garrison et al., 1984). In this study, three soybean physiological indexes the shoot *P*_N_ and root RA and RBSM had all shown a decreasing trend from the R2 to R6 stage in self-grafted parental (Dp), second-generation (Ds) and current (Dc) cultivars (Figure 2A,B, 3A), which matched to the theory described above. These results had also demonstrated that the soybean grafting technique did not cause any physiological abnormality which would disrupt shoot-root interactions and therefore was applicable to shoot-root interaction studies.

Firstly, the shoot *P*_N_ was examined in self-grafts, and it was found that current cultivars showed higher *P*_N_ than both parental and second-generation cultivars but only during the R6 stage (Figure 3A). This improvement of photosynthetic capacities was believed to be caused by extended active-growth periods in current cultivars (Rowntree et al., 2013; Keep et al., 2016; Koester et al., 2016). On the other hand, notably stronger levels of root RA and RBSM were observed during all R2, R5, and R6 stages in current cultivars compared to parental and second-generation cultivars (Figure 2A,B). Since most breeders often target for shoot traits and rarely consider roots during the breeding process, this root activity improvement has likely happened from the shoot improvement indirectly, which could evidence the utilization of shoot-root interactions in soybean production practices.

To better understand the causative relationship of positive correlations between *P*_N_ with root RA and RBSM, we then analyzed grafts with L14/Z35 scions and different year-released rootstocks for changes in *P*_N_. Surprisingly, current rootstocks could cause about 30% higher *P*_N_ during the R6 stage in the same record-yield scions compared with rootstocks of parental and second-generation cultivars (Figure 2C,D), which was consistent with our previous findings that photosynthetic traits in recently released cultivars could be further increased if root functions improved (Li et al., 2017b). Taken together, we can conclude that soybean roots influence the shoot photosynthetic characteristics. Prolong grain filling period is an important factor for yield potential determination (Minchin et al., 1980). During seed filling stage, plants begin senescencing which is accompanied by nitrogen (N) transfer from vegetative organs to the seeds. However, nearly 40% of N and phosphorus (P) in the pod is provided by the root system during this stage (Hanway and Weber, 1971). Liu et al. (2010) have compared the root senescence of soybeans with different yields at the seed filling stage and showing that the root senescence process of high-yielding cultivars was significantly lower than that of low-yielding cultivars. Therefore, the delay of root senescence and the enhancement of root absorption capacity during late seed filling stage could be particularly efficient yield improving strategies. Since the root system absorbs nutrients and water to fuel plant growth, stronger root physiological activities would become especially important for plants to retain photosynthetic capacities when the leaf senescence process is accelerating during late stages of the living cycle. This may explain why this positive influence on soybean *P*_N_ by the root activity was only observed during the R6 stage. From another perspective, strong root activities could also indirectly help with balancing shoot hormone levels which are essential for the final yield formation. Therefore, high R6 *P*_N_ can serve as an advantageous trait for future soybean high-yielding breeding programs.

Furthermore, our data had demonstrated that the soybean shoot could influence RA and root architecture. When grafted with different year-released scions, L14/Z35 rootstocks exhibited very similar patterns of RA (Figure 3B,C), root length and dry mass (Figure 4C–F) to those in the parental, second-generation and current controls. In addition, these rootstock differences were very obvious even at the earlier R2 stage when *P*_N_ were almost the same among different cultivars (Figure 3). Therefore, this soybean shoot to root influence seemed to be independent of photosynthetic characteristics. A large body of work has shown that signals travel long-distance to transmit messages between the plant shoot and root (Sharp, 2002; Choi et al., 2014; Olaetxea et al., 2015; Wege et al., 2015). For example, shoot-derived auxin could promote primary root elongation in arabidopsis (Sassi et al., 2012). A more recent study, which also adopted reciprocal grafting experiments with different species including *Vicia* *faba*, *Zea* *mays* and *Helianthus* *annuus*, had demonstrated that shoot-derived ABA could also function in promoting root growth (McAdam et al., 2016). Hence, a hypothesis could be drawn that certain soybean shoot-derived signaling molecules such as hormones influence the root activity and morphology. Nevertheless, detailed studies are still required to be conducted in order to pinpoint identities of signaling molecules which mediate the soybean shoot influences on root physiological functions.

### Soybean Yield

The most meaningful outcome of a crop plant system is its final yield. Various plant physiological processes are known to be yield-controlling factors such as the “source-sink” interaction. To achieve yield improvement, breeders often select cultivars with enhanced source and sink capacities. Older soybean cultivars were reported to be source-limited, and newer cultivars were more inclined toward being sink-limited (Liu et al., 2006). However, mechanistic understanding of the soybean yield formation especially from the “above-below ground” interaction perspective is valuable and yet to be achieved. Therefore, the ultimate goal of this study was to gain insights on how shoot-root interactions influence yield, which could help to reach extra yield gain in soybean production.

Overview examination of graft yield changes revealed that over ∼15% yield increase could be achieved in all five current scions when they were grafted onto L14/Z35 rootstocks, while only two out of six parental/second-generation scions (Amsoy and Kottman) showed comparable yield increases (Table 5). Among grafting combinations, the record-yield L14/Z35 scions exhibited quite stable yielding on current rootstocks and an obvious tendency of yield-loss on parental/second-generation rootstocks (such as ∼12% and ∼15% yield loss for Williams and Amsoy, respectively) (Table 5). Clearly soybean roots could contribute to the yield formation. Since this “root-to- yield” contribution had to be realized through the yield-controlling machinery in the shoot, the shoot-root compatibility appeared to be highly influential to soybean yield. In this aspect, current cultivars performed as superior matches with L14 and Z35 than parental and second-generation cultivars.

### Soybean Yield Formation by Components

Crop yield is formed comprehensively by yield components. Previous studies have suggested that increases in node m^-2^, reproductive node m^-2^, pod m^-2^, and seed m^-2^ and seed size/weight contributed sequentially to higher yields in more recently-bred Chinese and the United States soybean cultivars (Frederick et al., 1990; Cui and Yu, 2010; Kahlon et al., 2011). Another study with Midwestern United States soybean cultivars had shown that the yield increase was more strongly associated with SN than with seed size/weight (SS/SW) (Bruin and Pedersen, 2009). In soybean, SN-determination happens after booming and during early pod developmental stages. While SW is determined by SS potential and the degree of seed filling during late pod developmental stages. To our knowledge, there exists a SN-SW trade-off relationship since only limited resources are distributed between SN and SW during the whole grain setting periods. For example, higher SN often is accompanied with lower SW, or *vice versa* in current soybean cultivars (Supplementary Figure S1). In the present study, in order to gain a more detailed understanding on yield changes after grafting, SN and SW were chosen as yield-forming components and carefully examined from the shoot-root interaction perspective.

As we discussed earlier, total of seven common scions showed significant yield increase when grafted onto record-yield L14/Z35 rootstocks. Among them, yield increases were realized solely by SN-increase in Kottman and Dilworth; solely by SW-increase in Amsoy, Liaodou11 and 12; and additively by SN-and-SW-increase in Dennison and Tiefeng31 (Table 5). When these cultivars were cross-compared with the “map of genetic relationship of soybean cultivars”, it was very interesting to find that SN-increase happened in Williams-descendant cultivars and SW-increase in Amsoy-descendants (Figure 1). Presumably, genetic marks in Williams for this SN-increase-potential must have been transmitted to its progeny generations during decades of breeding and the same scenario should also apply to the SW-increase-potential in Amsoy. These speculations could be confirmed in Dennison grafts. Since it contained bloodlines from both Williams and Amsoy, as expected it showed both SN-and SW-increasing phenotypes. However, no SW-increase was observed in grafts with Kottman scions (Table 5 and Figure 1), which supposedly should also contain Amsoy bloodline. A possible explanation was that although the superior L14/Z35 root function was able to increase the total yield potential of Kottman scion to certain extent it was not enough to support extra SW-increase on the top of over 30% of SN-increase. Nevertheless, these results suggested that soybean roots were capable of influencing yield-forming components but in a shoot-genotype-specific manner.

The fact that only Williams, Amsoy, and Liaodou10 rootstocks caused relatively mild yield-loss in the record-yield L14 and Z35 scions reflected the high flexibility of L14 and Z35 in utilization of root support to realize their yield potentials. On the other hand, a tendency of SN-decrease accompanied by SW-increase was noticeable with eight out of eleven common rootstocks (Table 6). L14 and Z35 were two cultivars with very high SN-potentials (Supplementary Figure S1). Therefore, it was likely that most common rootstocks were not able to support such high SN-potentials so that caused SN-decreases. However, the quite stable yield potentials in L14 and Z35 still had to be distributed into SW, consequently reflected into the SW-increase phenotype. In addition, another observation deserved special attention. An opposite tendency of SN- increase and SW-decrease was identified in grafts with Kottman, Dilworth and Dennison rootstocks (Table 6). Although the data seemed scratchy and no clear conclusion could be made, it clearly showed an example of boosting the already strong SN trait in L14 and Z35 to a higher level by grafting with other rootstocks.

## Conclusion

Taken together, soybean shoot-root interactions could be pinpointed to the photosynthesis-root activity association and yield-formation determination processes. Soybean roots should play supportive rather than decisive roles in shoot-root interactions. Strong root activity was advantageous for the grain-filling stage and probably the SW-formation through photosynthetic pathways. Since soybean SN- determination happened before the grain-filling stage, root-influence on SN- determination might be mediated through mechanisms such as nutrition absorption and distribution, because studies have shown that fertilization could significantly increase SN in current Chinese and the United States soybean cultivars (Guo et al., 2015). In order to reveal more comprehensive and detailed “cause and effect” relationships of soybean shoot-root interactions in the future, grafting should be adopted as a powerful tool to be combined with available genetic and genomic technological platforms.

During past soybean breeding programs, there was an uncoordinated improvement between roots and yields (Li et al., 2018). Selecting suitable root systems, which could match the shoot yield potentials, is important for future soybean breeding. Two breeding strategies should be proposed to gain extra yield in current cultivars by root improvement: to increase SN in high-SW cultivars and to increase SW in high-SN cultivars. In the present study, Tiefeng31 and Dennison were such cultivars with the proper yield-formation characteristics for these breeding strategies (Supplementary Figure S1). Their success in the yield-improvement had provided confidence for wider adoption of the SN- and SW-increasing strategies in future breeding programs (Supplementary Figure S1 and Table 4). Finally, grafting had been proved to be a valuable technique which would accelerate the searching for optimal parent combinations in future soybean high-yielding breeding efforts.

## Author Contributions

FX and MZ designed the study. YD, QZ, SL, and XY performed all the experiments. YD and QZ analyzed the data. YD, FX, and MZ discussed the data. YD wrote the manuscript.

## Conflict of Interest Statement

The authors declare that the research was conducted in the absence of any commercial or financial relationships that could be construed as a potential conflict of interest.
