# Supplementary material for: Shoot/Root Interactions Affect Soybean Photosynthetic Traits and Yield Formation: A Case Study of Grafting With Record-Yield Cultivars
Source: Front Plant Sci. 2019 Apr 9;10:445. doi: 10.3389/fpls.2019.00445 (PMC6465614; doi:10.3389/fpls.2019.00445)
Supplement: Supplementary file 1 [file Table_1.docx]

Supplementary Material

Shoot/root interactions affect soybean photosynthetic traits and yield formation: a case study of grafting with record-yield cultivars

Yanli Du, Qiang Zhao, Shengyou Li, Xingdong Yao, Futi Xie*, Mingzhe Zhao*

*** Correspondence:** Futi Xie: snsoybean@sohu.com

Mingzhe Zhao: mzhao718@hotmail.com

## Supplementary Figures

**Fig. S1 Seed number and seed weight (per hundred seeds) of self-grafted the USA and Chinese cultivars. Wlm: Williams; Ams: Amsoy; L10: Liaodou10; T31: Tiefeng31; L11: Liaodou11; L3: Liaodou3; Rsk: Resnik; L12: Liaodou12; Ktm: Kottam; Dlw: Dilworth; Dnn: Dennison; L14: Liaodou14; Z35: Zhonghuang35.**

## Supplementary Tables

**Table S1 the USA and Chinese soybean cultivars for grafting**

**Table S2 *Pearson’s* correlation coefficients between photosynthetic and root architectural indexes during reproductive stages in 2014 and 2015**

**

**

**Fig. S1 Seed number (A) and seed weight (per hundred seeds) (B) of self-grafted the USA and Chinese cultivars. Wlm: Williams; Ams: Amsoy; L10: Liaodou10; T31: Tiefeng31; L11: Liaodou11; L3: Liaodou3; Rsk: Resnik; L12: Liaodou12; Ktm: Kottam; Dlw: Dilworth; Dnn: Dennison; L14: Liaodou14; Z35: Zhonghuang35.**

**Table S1 the USA and Chinese soybean cultivars for grafting**

| Group | Cultivar | Growth habit | Ancestor | Breeding Location | Year of release | Pedigree |
| --- | --- | --- | --- | --- | --- | --- |
| Common parents (Dp) | Amsoy | Indeterminate | - | Iowa State, USA | 1966 | Adams × Harosoy |
|  | Williams | Indeterminate | - | USDA, USA | 1971 | Wayne × L57-0034 |
|  |  |  |  |  |  |  |
| Second-generation cultivars (Ds) | Liaodou 3 | Semi-determinate | Amsoy | Liaoning, CN | 1983 | Tiefeng18 × Amsoy |
|  | Liaodou 10 | Semi-determinate | Amsoy | Liaoning, CN | 1991 | Liaodou 3 × Liao 82-5185 |
|  | Resnik | Indeterminate | Williams | Ohio State, USA | 1987 | A3127 × L24 |
|  | Kottman | Indeterminate | Amsoy, Williams | Ohio State, USA | 1991 | HS88-7363× HS88-4988 |
|  |  |  |  |  |  |  |
| Current cultivars (Dc) | Liaodou 11 | Semi-determinate | Amsoy | Liaoning, CN | 1996 | Liao 84063 × Liaodou 3 |
|  | Liaodou 12 | Semi-determinate | Amsoy | Liaoning, CN | 2001 | Liao 85094 × Liaodou 10 |
|  | Tiefeng 31 | Semi-determinate | Williams | Tieling, CN | 2001 | Xin 3511 × Resnik |
|  | Dilworth | Indeterminate | Williams | Ohio, USA | 2002 | Chapman × Probst |
|  | Dennison | Indeterminate | Amsoy, Williams | Ohio, USA | 2006 | Athow × HS94-4533 |
|  |  |  |  |  |  |  |
| Record-yield | Liaodou14  (L14) | Semi-determinate | - | Liaoning, CN | 2003 | Liaodou10 × Mecury |
|  | Zhonghuang35  (Z35) | Determinate | - | Beijing, CN | 2006 | (PI486355 × Zheng 8431)  × Zheng 6062 |

**Table S2 *Pearson’s* correlation coefficients between photosynthetic and root architectural indexes during reproductive stages in 2014 and 2015**

|  |  | Year of 2014 | | | | | |  | Year of 2015 | | | | | |
| --- | --- | --- | --- | --- | --- | --- | --- | --- | --- | --- | --- | --- | --- | --- |
|  |  | R2 | | R5 | | R6 | |  | R2 | | R5 | | R6 | |
|  |  | Length | Dry mass | Length | Dry mass | Length | Dry mass |  | Length | Dry mass | Length | Dry mass | Length | Dry mass |
| D/D | *P*_N_ | 0.02 ^NS^ | 0.16 ^NS^ | 0.14 ^NS^ | 0.21 ^NS^ | 0.66 ** | 0.42 * |  | -0.01 ^NS^ | 0.23 ^NS^ | -0.02 ^NS^ | 0.12 ^NS^ | 0.01 ^NS^ | 0.29 ^NS^ |
|  | *g*_s_ | -0.09 ^NS^ | -0.12 ^NS^ | -0.21 ^NS^ | 0.02 ^NS^ | 0.66 ** | 0.62 ** |  | 0.32 ^NS^ | 0.32 ^NS^ | 0.00 ^NS^ | -0.03 ^NS^ | 0.12 ^NS^ | 0.24 ^NS^ |
|  | *E* | 0.15 ^NS^ | 0.06 ^NS^ | 0.20 ^NS^ | 0.46 ** | 0.41* | 0.28 ^NS^ |  | -0.02 ^NS^ | -0.13 ^NS^ | 0.37 * | 0.36 * | 0.20 ^NS^ | 0.00 ^NS^ |
|  | Leaf greeness | 0.15 ^NS^ | 0.19 ^NS^ | -0.19 ^NS^ | -0.06 ^NS^ | 0.22 ^NS^ | 0.20 ^NS^ |  | 0.26 ^NS^ | 0.18 ^NS^ | -0.23 ^NS^ | -0.14 ^NS^ | -0.15 ^NS^ | 0.23 ^NS^ |
|  |  |  |  |  |  |  |  |  |  |  |  |  |  |  |
| L/D | *P*_N_ | 0.67 ** | 0.31 ^NS^ | -0.06 ^NS^ | -0.07 ^NS^ | 0.87 ** | 0.77** |  | 0.38 * | 0.53 ** | -0.01 ^NS^ | -0.04 ^NS^ | 0.73 ** | 0.79 ** |
|  | *g*_s_ | 0.29 ^NS^ | 0.20 ^NS^ | -0.27 ^NS^ | -0.14 ^NS^ | 0.70 ** | 0.64 ** |  | -0.12 ^NS^ | 0.52 ** | -0.18 ^NS^ | -0.09 ^NS^ | 0.60 ** | 0.70 ** |
|  | *E* | 0.55 ** | 0.08 ^NS^ | 0.13 ^NS^ | 0.25 ^NS^ | 0.62 ** | 0.63 ** |  | -0.23 ^NS^ | 0.50 ** | 0.17 ^NS^ | 0.19 ^NS^ | 0.58 ** | 0.62 ** |
|  | Leaf greeness | 0.69 ** | 0.27 ^NS^ | 0.15 ^NS^ | 0.10 ^NS^ | 0.33 ^NS^ | 0.19 ^NS^ |  | 0.22 ^NS^ | 0.27 ^NS^ | 0.13 ^NS^ | 0.03 ^NS^ | 0.24 ^NS^ | 0.33 ^NS^ |
|  |  |  |  |  |  |  |  |  |  |  |  |  |  |  |
| Z/D | *P*_N_ | 0.43 * | -0.19 ^NS^ | -0.19 ^NS^ | -0.28 ^NS^ | 0.85 ** | 0.78 ** |  | 0.14 ^NS^ | 0.42 * | 0.52 ** | 0.55 ** | 0.82 ** | 0.90 ** |
|  | *g*_s_ | 0.11 ^NS^ | -0.21 ^NS^ | -0.10 ^NS^ | 0.01 ^NS^ | 0.44 * | 0.32 ^NS^ |  | 0.18 ^NS^ | 0.08 ^NS^ | -0.02 ^NS^ | 0.04 ^NS^ | 0.48 ** | 0.47 ** |
|  | *E* | 0.11 ^NS^ | -0.40 * | 0.02 ^NS^ | 0.15 ^NS^ | 0.56 ** | 0.62 ** |  | 0.11 ^NS^ | 0.20 ^NS^ | 0.57 ** | 0.66 ** | 0.50 ** | 0.51 ** |
|  | Leaf greeness | -0.02 ^NS^ | 0.06 ^NS^ | -0.15 ^NS^ | -0.12 ^NS^ | 0.06 ^NS^ | 0.10 ^NS^ |  | 0.16 ^NS^ | 0.36 * | 0.44 ** | 0.57 ** | 0.32 ^NS^ | 0.38 * |
|  |  |  |  |  |  |  |  |  |  |  |  |  |  |  |
| D/L | *P*_N_ | -0.23 ^NS^ | -0.19 ^NS^ | 0.28 ^NS^ | 0.34 ^NS^ | 0.71** | 0.30 ^NS^ |  | 0.28 ^NS^ | 0.05 ^NS^ | -0.14 ^NS^ | 0.18 ^NS^ | -0.07 ^NS^ | 0.18 ^NS^ |
|  | *g*_s_ | -0.00 ^NS^ | -0.11 ^NS^ | -0.29 ^NS^ | -0.04 ^NS^ | 0.54 ** | 0.36 * |  | 0.24 ^NS^ | 0.25 ^NS^ | -0.09 ^NS^ | -0.11 ^NS^ | -0.19 ^NS^ | 0.03 ^NS^ |
|  | *E* | -0.04 ^NS^ | -0.28 ^NS^ | -0.08 ^NS^ | 0.18 ^NS^ | 0.24 ^NS^ | 0.29 ^NS^ |  | -0.02 ^NS^ | -0.00 ^NS^ | 0.09 ^NS^ | 0.03 ^NS^ | 0.15 ^NS^ | 0.00 ^NS^ |
|  | Leaf greeness | -0.18 ^NS^ | -0.15 ^NS^ | 0.00 ^NS^ | 0.15 ^NS^ | 0.26 ^NS^ | 0.17 ^NS^ |  | 0.27 ^NS^ | -0.17 ^NS^ | 0.01 ^NS^ | -0.04 ^NS^ | -0.57 ** | 0.31 ^NS^ |
|  |  |  |  |  |  |  |  |  |  |  |  |  |  |  |
| D/Z | *P*_N_ | -0.01 ^NS^ | -0.01 ^NS^ | -0.11 ^NS^ | 0.01 ^NS^ | 0.49 ** | 0.22 ^NS^ |  | 0.43 * | 0.42 * | -0.14 ^NS^ | -0.00 ^NS^ | 0.52 ** | 0.60 ** |
|  | *g*_s_ | -0.21 ^NS^ | -0.22 ^NS^ | -0.23 ^NS^ | -0.06 ^NS^ | 0.29 ^NS^ | 0.25 ^NS^ |  | 0.15 ^NS^ | 0.15 ^NS^ | -0.34 ^NS^ | -0.22 ^NS^ | 0.29 ^NS^ | -0.00 ^NS^ |
|  | *E* | -0.23 ^NS^ | -0.26 ^NS^ | 0.39 * | 0.32 ^NS^ | 0.22 ^NS^ | 0.17 ^NS^ |  | 0.18 ^NS^ | 0.09 ^NS^ | -0.10 ^NS^ | -0.21 ^NS^ | 0.38 * | 0.08 ^NS^ |
|  | Leaf greeness | 0.107 ^NS^ | 0.295 ^NS^ | 0.180 ^NS^ | 0.243 ^NS^ | -0.05 ^NS^ | -0.086 ^NS^ |  | 0.050 ^NS^ | 0.089 ^NS^ | -0.037 ^NS^ | 0.132 ^NS^ | 0.058 ^NS^ | 0.519** |

**R2: the flowering stage; R5: early grain-filing stage; R6: late grain-filling stage. D: mean of eleven common cultivars; D/D: self-grafts; L/D or Z/D: grafts with L14/Z35 scions; D/L or D/Z: grafts with L14/Z35 rootstocks. *P*_N_: net photosynthetic rate; *g*_s_: stomatal conductance; *E*: transpiration rate. RA: root activity; RBSM: root bleeding sap mass. * *P* < 0.05, ** *P* < 0.01, NS: not significant.**
